# Effect of oral health on daily performance among Indian teachers

**DOI:** 10.6026/973206300210918

**Published:** 2025-05-31

**Authors:** Chanchal Gangwar, Pallavi Patel, Akshay J. Melath, Kritika Saxena, Pooja Pani, Sonika Singh, Anukriti Kumari

**Affiliations:** 1 Department of Public Health Dentistry, Institute of Dental Sciences, Bareilly, Uttar Pradesh, India; 2 Department of Periodontology, Guru Gobind Singh college of Dental Science and Research Centre, Burhanpur, Madhya Pradesh, India; 3 Department of Health Science, Uttranchal University, Dehradun, Uttrakhand, India; 4 Department of Pediatric and Preventive Dentistry, Awadh Dental College and Hospital, Jamshedpur, Jharkhand, India; 5 Department of Public Health Dentistry, Teerthankar Mahaveer Dental College & Research Centre, Moradabad, Uttar Pradesh, India; 6 Department of Oral Medicine and Radiology, School of Dental Sciences, Sharda University, Greater Noida, India

**Keywords:** Dental caries, oral health-related quality of life (OHRQoL), oral impact on daily performance (OIDP), oral health, teachers

## Abstract

Oral health-related quality of life (OHRQoL) using the oral impact on daily performance (OIDP) instrument among secondary school teachers in India is of interest. Hence, a cross-sectional survey of 1600 teachers aged 24-57 participated in interviews and oral health examinations. About 59.5% reported oral health issues affecting daily activities, with eating and sleeping most impacted. The OIDP showed high reliability (Cronbach's alpha = 0.87) and significant validity (p<0.05). Thus, OIDP is a reliable tool for evaluating OHRQoL in this population.

## Background:

WHO (1948) defined health as "a state of complete physical, mental, and social well-being, not merely the absence of disease or infirmity," which remains relevant today. Oral health, an integral part of overall health, affects daily activities such as eating, laughing, speaking, and maintaining one's appearance. Although disease and health often overlap, they are not experienced simultaneously, and perceptions of health and disease may vary among individuals [1]. Health-related quality of life (HRQoL) encompasses the physical, psychosocial, and social aspects of daily life and the impact of disease on an individual's functioning, as perceived by the individual. Oral health-related quality of life (OHRQoL) is a multidimensional concept related to oral health and disease, with studies showing that poor oral health significantly impacts daily life, particularly among the elderly and those who do not regularly utilize dental services [2]. Research has highlighted weak relationships between clinical indicators of oral disease and OHRQoL, indicating a discrepancy between professionally assessed quality of life and self-rated oral health status [3, 4]. The oral impact on daily performance (OIDP) inventory assesses the frequency and severity of daily performance impacts; however, unweighted frequency scores have proven sufficient for practical use, as applied in this study. Teachers play a crucial role in shaping not only the academic lives of children but also in promoting their overall well-being. Through their influence, knowledge, and regular interaction with students, teachers can contribute effectively to students' health education when adequately trained and supported [5]. Despite this, very few studies have assessed OHRQoL among school teachers. Therefore, it is of interest to determine OHRQoL among secondary school teachers in India using the OIDP instrument, acknowledging that their perceptions of health and disease directly impact their quality of life.

## Methodology:

A descriptive, cross-sectional survey was conducted to assess the psychometric properties of the OIDP inventory among Secondary School Teachers in India. Sample size estimation was conducted based on the prevalence of "Difficulty with Eating and Enjoying Food," which was 52%, as reported in a study conducted among adults in India. Based on that prevalence, the sample size for the present study was calculated using the following formula:-

N= 4Pq/L2

Where,

Prevalence of "Difficulty with Eating and Enjoying Food," which was 52%

q= (1-P)

= (100-52)

= 48%

Allowable error in the prevalence (precision)

= 5 % of the P

= 52 x 5/100

= 2.5

According to the formula (After substitution), the sample size was determined to be:

N = 4 X 52 X 48/2.5 X 2.5

=1597 (Calculated sample size)

= 1600 (Final sample size)

The sample size was rounded to 1,600 secondary school teachers.

We selected 127 secondary schools, comprising 77 Government and 50 private schools, in India. Schools were broadly categorized into Government and Private aided, thereby providing an opportunity to incorporate teachers from diverse backgrounds. In each school, approximately 15-30 teachers were employed, with the number of teachers varying depending on the school's student enrollment. India was arbitrarily divided into four zones: North, East, West, and South. From each zone, 10 Government schools and 10 Private schools were selected using the Simple Random Sampling method (Also Known as the Lottery method). Approximately 20 School teachers from each school were chosen using a lottery method to participate in the study. In schools where the total number of teachers was higher, additional teachers (More than 20) were included to compensate for the fewer teachers available in some schools. Ultimately, a total of 400 teachers were selected from each zone to achieve the desired sample size.

## Examiner's schedule:

Data collection occurred between January 15 and March 30, 2024, excluding Sundays and holidays. Schools were informed of the schedule one month in advance, with flexibility to accommodate any delays. A minimum of 20 teachers were interviewed and examined daily by a single trained and calibrated examiner (CG). A pilot study on 50 teachers was conducted to assess feasibility, and Kappa values of 0.81 (intra-examiner) and 0.83 (inter-examiner) indicated high reliability. Informed written consent was obtained from all participants.

## Study proforma:

The proforma was written in English and consisted of two parts: a questionnaire and a clinical examination. The questionnaire collected demographic data, oral health behaviors, OIDP inventory responses, and perceptions of general and oral health. The OIDP inventory assessed eight aspects of daily performance, with responses recorded on a 5-point scale.

## OIDP inventory and scoring:

OIDP scores assessed the frequency and severity of oral health impacts on daily performances over the past six months. Simple Count (SC) and Additive (ADD) scores were calculated, with SC reflecting affected performances and ADD measuring cumulative impact. Responses on general and oral health were coded on a 3-point scale.

## Validity and oral examination:

Construct validity was assessed using perceived oral health and general health, while criterion validity was evaluated using caries experience as a proxy. Oral examinations assessed decayed, missing, and filled teeth (DMFT Index) under adequate illumination. The inclusion criteria included English proficiency and consent, while participants who were unwilling or absent during the study were excluded.

## Data analysis:

The data were analyzed using SPSS version 21, with a significance level set at 0.05%. Descriptive analysis covered demographic profiles, oral health habits, OIDP prevalence, and satisfaction with dental appearance. Reliability was assessed using Cronbach's alpha, and non-parametric tests (Kruskal-Wallis) were used for validity and prevalence analysis.

## Results:

A total of 1600 Secondary School Teachers in India, 821 (51.3%) males and 779 (48.7 %) females aged 24 - 57 years (mean 39.6 ± 8.04), participated in the study. The majority (42%) of the teachers belonged to the 31-40 age group. Most of the school teachers (92.2 %) were graduates. In the present study, 51.3% of the school teachers belonged to private schools, and 48.7% belonged to government schools, respectively (Table 1). All the school teachers in the study used toothpaste and a toothbrush for cleaning their teeth. Most school teachers (65.2%) brushed their teeth only once a day and only 10.5% had a habit of rinsing their mouths after every meal. The bulk of the school teachers (86.1%) visited the dentist either every six months or once a year for regular check-ups. Table 1 gives the prevalence of oral impacts among the school teachers. The most prevalent effect was 'on eating', followed by sleeping and relaxing, cleaning of teeth, and speaking clearly in the past six months. The mean OIDP SC score for the study population was 2.48 ± 2.49 (Range: 0 -8) (Table 2). Table 2 shows reliability analysis using Cronbach's alpha. The OIDP instrument demonstrated good reliability and showed homogeneity of items, with a Cronbach's alpha of 0.87. All corrected item total correlations were above the minimum recommended level of 0.20 to be included in the scale. All were positive, and no correlation was sufficiently high for any item to be considered redundant. For the OIDP performance scores, the inter-item correlation coefficients ranged between 0.02 (relationship between cleaning teeth and carrying out work) and 0.65 (relationship between eating, sleeping, and relaxing) (Table 3). Construct validity of the instrument was demonstrated in that the mean OIDP scores showed a clear trend with OIDP scores; those with perceived 'dissatisfaction with general health', 'oral health ' and 'dental appearance' having higher OIDP score, indicating higher level of oral impacts (p<0.05) (Table 4). The mean DMFT among the participants was 3.00 ± 1.67 (DT = 1.48 ± 1.48, MT = 0.55 ± 0.85, FT = 0.98 ± 1.53). For criterion validity, as the number of decayed and missing teeth increased, the mean impact score also increased and was found to be statistically significant (p < 0.05) (Table 5). Age was found to be statistically associated (p < 0.05) with mean OIDP SC scores, indicating that older teachers perceived oral health as having a more significant impact on their quality of life compared to their younger contemporaries (Table 6). School teachers who visited the dentist only during distress (Others) had significantly higher mean OIDP SC scores compared to those who visited the dentist regularly or annually, and this difference was found to be statistically significant (p < 0.05) (Table 7).

## Discussion:

Over the past two decades, various instruments integrating subjective and objective oral health measures have been developed [6]. This study was the first to apply the OIDP inventory to assess OHRQoL among secondary school teachers. OIDP was selected due to its strong psychometric properties and logical approach to quantifying impacts, which evaluates both frequency and severity [7]. As participants were familiar with English, no translation was needed, avoiding rigorous back-translation [8, 9]. All participants had visited a dentist at least once, with 86.1% visiting biannually or annually, reflecting a positive attitude towards oral health. This contrasts with studies from Pakistan, where one-third of teachers never visited a dentist [10], and Nigeria, where only 42.4% had ever visited a dentist [11]. Cronbach's alpha was 0.87, indicating excellent reliability, consistent with previous OIDP applications that reported values between 0.67 and 0.90 [12]. The prevalence of oral impacts was 60%, comparable to studies in Uganda (62%) [13], Iran (64%) [14] and Korea (62%) [15], but lower than studies in India (72%) [16]. Variations may arise from different populations, disease levels, and socio-cultural perceptions. The most affected daily performance was eating and enjoying food (60%) and this is consistent with other studies on OIDP [17, 18]. The mean DMFT ranged from 0 to 9 (mean 3.0, SD 1.67), with 61% of individuals having decayed teeth and 37% having missing teeth, indicates a high level of unmet treatment needs. A significant association was observed between OIDP scores and self-rated general health, oral health, and satisfaction with dental appearance (p < 0.05), suggesting excellent construct validity [16, 18]. Criterion validity was demonstrated by the OIDP's ability to discriminate between participants with varying decayed and missing teeth, consistent with other studies [14, 15, 16, 17- 18]. Participants aged 41-50 and those over 50 years experienced more significant oral health impacts (p = 0.01), consistent with findings from Thailand [17]. Regular dental visits had a reduced impact on oral health compared to visits made during pain or discomfort (p = 0.00), aligning with other OIDP studies. In contrast to the studies by Kumari *et al.* [19] and Dhama *et al.* [20], which focused on broader populations including adolescents and rural adults, our study uniquely targeted secondary school teachers relatively underexplored occupational group. While Kumari *et al.* reported lower oral health-related impacts using the OHIP-14 tool, our study observed a 60% prevalence of impacts using the OIDP, particularly affecting eating and relaxing. Dhama *et al.* highlighted poor oral hygiene practices and limited dental visits, whereas our participants demonstrated a more positive oral health attitude, with 86.1% visiting the dentist regularly. Additionally, our study showed a stronger association between OIDP scores and self-rated oral health, validating the instrument's utility in educated populations. These comparisons underscore the importance of context, occupation, and assessment tools in evaluating OHRQoL outcomes.

## Study limitations:

Despite ensuring validity through calibration, duplicate examinations, and rigorous training, the cross-sectional design limits analytical capability. Since OIDP relies on participants' perceptions, memory-based responses may underestimate functional and psychosocial impacts.

## Conclusion:

The OIDP inventory was used to assess the impact of oral health on daily activities and has proven to be a valid and reliable measure of OHRQoL across various cultural settings. It should also be applied in clinical settings to assess its usefulness in individual treatment planning and evaluating treatment outcomes. Thus, the need to move beyond normative assessments and incorporate OHRQoL measures into oral health care services is emphasized.

## Figures and Tables

**Table 1:** Prevalence of components of OIDP inventory (ADD Score) among school teachers

Parameters of Oral Impact on Daily	Every/nearly every day	Three to four times a week	Once or twice a week	Less than once a month	Total Affected	Never affected
Performance (OIDP) Instrument	N/ (%)	N/ (%)	N/ (%)	N/ (%)	N/ (%)	N/ (%)
Eating and enjoying food	0/(0)	8/(0.6)	20/(1.4)	926/(57.5)	954/(59.5)	646/(40.5)
Speaking and pronouncing clearly	0/(0)	8/(0.5)	95/ (5.9)	408/ (25.5)	511/ (31.9)	1089/ (68.1)
Cleaning teeth	0/(0)	20/ (1.3)	120/ (7.5)	427/(26.7)	567/ (35.6)	1033/ (64.4)
Sleeping and relaxing	0/(0)	16/ (1.0)	172/ (10.8)	603/ (37.7)	791/ (49.4)	809/ (50.6)
Smiling and showing teeth without embarrassment	0/(0)	0/ (0)	59/ (3.7)	322/ (20.1)	381/(23.8)	1219/ (76.2)
Maintaining a usual emotional state	0/(0)	0/ (0)	16/ (1.0)	291/ (18.2)	307/ (19.2)	1293/ (80.8)
Carrying out major work and a social role	0/(0)	0/ (0)	0/(0)	151/ (9.4)	151/ (9.4)	1449/ (90.6)
Enjoying contact with people	0/(0)	0/ (0)	0/(0)	232/ (14.5)	232/ (14.5)	1368/ (85.5)

**Table 2:** Reliability analysis using Cronbach's alpha in the study population

OIDP Items	Corrected Item-Total Correlation	Cronbach's Alpha if the item is deleted
Eating and enjoying food	0.62	0.85
Speaking and pronouncing clearly	0.75	0.83
Cleaning teeth	0.52	0.86
Sleeping and relaxing	0.59	0.85
Smiling and showing teeth without embarrassment	0.59	0.85
Maintaining a usual emotional state	0.68	0.84
Carrying out major work and a social role	0.56	0.85
Enjoying contact with people	0.65	0.84
Standardized Cronbach's alpha	0.87	

**Table 3:** Reliability analysis of OIDP index for secondary school teachers: OIDP items correlation matrix

Performance Scores	Eating and Enjoying Food	Speaking and Pronouncing Clearly	Cleaning Teeth	Sleeping and Relaxing	Smiling and showing teeth without embarrassment	Maintaining a usual emotional state	Carrying out major work and a social role	Carrying out major work and a social role
Eating and Enjoying Food	1							
Speaking and Pronouncing Clearly	0.56**	1						
Cleaning Teeth	0.49**	0.38**	1					
Sleeping and Relaxing	0.65**	0.56**	0.39**	1				
Smiling and showing teeth without embarrassment	0.36**	0.19**	0.36**	0.19**	1			
Maintaining a usual emotional state	0.36**	0.41**	0.28**	0.42**	0.50**	1		
Carrying out major work and a social role	0.22**	0.31**	0.02**	0.34**	0.33**	0.48**	1	
Enjoying contact with people	0.35**	0.47**	0.34**	0.45**	0.35**	0.49**	0.71**	1
Pearson's correlation coefficient test
** All correlations statistically significant at the 0.01 level (2-tailed)

**Table 4:** Construct validity for OIDP inventory

Variable	Responses	OIDP Mean ± SD	p value
Construct validity			
Perceived General Health Status	Satisfied	10.65 ± 2.80	0.018*
	Average	10.82 ± 2.88	
	Dissatisfied	0 .0 ± 0.0	
Perceived Oral Health Status	Satisfied	10.60 ± 2.75	0.033*
	Average	10.82 ± 2.90	
	Dissatisfied	10.86 ± 2.90	
Perceived satisfaction with the appearance of teeth	Satisfied	10.64 ± 2.90	0.041*
	Average	10.69 ± 2.82	
	Dissatisfied	10.84 ± 2.88	
*p < 0.05 (S)

**Table 5:** Criterion validity for OIDP inventory

Variable	Responses	OIDP Mean ± SD	p value
Criterion validity			
Decayed teeth	0- 2.0	10.78 ± 2.853	0.04*
	3.0- 4.0	10.87 ± 2.978	
	5.0- 6.0	10.94 ± 2.964	
Missing teeth	0- 2.0	10.81 ± 2.88	0.05*
	3.0- 4.0	10.85 ± 2.91	
	5.0- 6.0	10.88 ± 2.95	
Filled teeth	0- 2.0	10.79 ± 2.883	0.12
	3.0- 4.0	10.86 ± 2.917	
	5.0- 6.0	10.76 ± 2.703	
DMFT	0- 2.0	10.75 ± 2.826	0.03*
	3.0- 4.0	10.85 ± 2.928	
	5.0- 6.0	10.80 ± 2.890	
	Above 6	10.67 ± 3.031	
*p < 0.05 (S)

**Table 6:** Assessment of age with mean OIDP simple count scores in the study population

Age	Number	Mean OIDP SC SCORE	p value
21-30 years	214	2.48 ± 2.40	0.018*
31-40 years	670	2.61 ± 2.54	
41-50 years	541	2.24 ± 2.46	
51 years & above	175	2.73 ± 2.45	
Total	1600	2.48 ± 2.49	
Kruskal-Wallis Test,
*p < 0.05 (S)

**Table 7:** Assessment of frequency of dental visit with mean OIDP simple count (SC) scores in study population

Frequency of Dental Visits	Number	Mean OIDP SC SCORE	p value
Regularly or every six months	699	2.19 ± 2.32	0.000*
Annually	679	2.56 ± 2.59	
Others	222	3.14 ± 2.54	
Total	1600	2.48 ± 2.49	
Kruskal-Wallis Test,
*p < 0.05 (S)
